# Expression levels and genetic polymorphisms of interleukin-2 and interleukin-10 as biomarkers of Graves’ disease

**DOI:** 10.3892/etm.2015.2180

**Published:** 2015-01-14

**Authors:** CUIGE LIANG, WENHUA DU, QINGYU DONG, XIAOMENG LIU, WENXIA LI, YUELI WANG, GUANQI GAO

**Affiliations:** Department of Endocrinology, Linyi People’s Hospital, Linyi, Shandong 276003, P.R. China

**Keywords:** interleukin-2, interleukin-10, biomarker, Graves’ disease, genetic polymorphism

## Abstract

The aim of the present study was to determine whether the expression levels of interleukin (IL)-2 and IL-10 may be used as biological markers in Graves’ disease (GD) patients. A total of 256 individuals, including 118 GD patients and 138 healthy individuals, were enrolled into the study. Blood samples were collected from each patient and healthy individual, which were then subjected to enzyme-linked immunosorbent assay (ELISA). Total RNA and total proteins were determined using reverse transcription-quantitative polymerase chain reaction (RT-qPCR) and western blot analysis, respectively. In addition, restriction fragment length polymorphism (RFLP) analysis was performed to detect the presence of genetic polymorphisms. The ELISA results indicated that the IL-2 and IL-10 serum levels in the GD patients were increased by ~5.2 and ~7-fold when compared with the levels in the healthy controls. The results of RT-qPCR indicated that the mRNA expression levels of IL-2 and IL-10 were upregulated in the GD patients when compared with the healthy controls. Furthermore, the western blot analysis results revealed that the protein expression levels of IL-2 and IL-10 were significantly increased in the GD patients. RFLP analysis indicated that the increased number of GG single nucleotide polymorphisms (SNPs) in the GD group were detected in the −330 locus of the IL-2 promoter and the −1082 locus of the IL-10 promoter. In addition, the results indicated that the relatively high rates of homozygous GG SNPs (IL-2 −330T/G and IL-10 −1082A/G polymorphisms) on the alleles may be associated with the incidence of GD. The serum, mRNA and protein expression levels of IL-2 and IL-10 were significantly increased in GD patients when compared with the levels in the healthy controls. In conclusion, the expression levels and genetic polymorphisms of IL-2 and IL-10 may be potential biomarkers for the incidence of Graves’ disease in the population studied.

## Introduction

Graves’ disease (GD) is an autoimmune disorder, which is caused by abnormal genetic alterations, including alterations in the expression levels of certain human cellular genes, and various environmental factors, such as smoking (1). GD mainly affects the thyroid, frequently resulting in enlarged and overactive thyroid glands, as well as several symptoms, including muscle weakness, insomnia and irritability (2,3). In addition, GD may affect the eyes, resulting in exophthalmos, or other systems of the body, including the skin, heart, circulation and nervous system. Up to 2% of the female population is affected by GD, with a female-to-male ratio between 5:1 and 10:1 (4,5). Furthermore, interactions between genetic factors and environmental factors may increase the risk of GD (6,7).

Previous studies have identified ~20 genetic polymorphisms that are associated with GD, including genes associated with the thyroid or involved in autoimmune responses (8,9). T helper type 1 (Th1) and Th2 serum cytokines have been demonstrated to be involved in the development of GD (10–12). Interleukin (IL)-2, a cytokine signaling molecule, is a protein regulating the activities of lymphocytes that are involved in immunity. IL-2 is required for the proliferation and differentiation of human T cells into effector cells (13). A previous study reported that the serum concentrations of IL-2 were increased in patients with vitiligo, which is an acquired depigmenting disorder associated with GD and characterized by the loss of functional melanocytes (14).

IL-10 is an anti-inflammatory cytokine, also known as a human cytokine synthesis inhibitory factor, which presents pleiotropic effects in inflammation and immunoregulation. IL-10 inhibits the expression levels of Th1 cytokines, major histocompatibility complex class II antigens and costimulatory molecules on macrophages (15). In addition, IL-10 assists B-cell survival, proliferation and antibody production and affects the functionality of certain cellular pathways. For instance, it inhibits the activities of nuclear factor-κB and alters the JAK-STAT signaling pathway (16). A previous study on a murine model indicated that IL-10 deficiency reduces the induction of anti-thyroid stimulating hormone receptor antibodies; thus, IL-10 plays an important role in the development of GD in mice (17).

In the present study, the serum levels of IL-2 and IL-10 were investigated in GD patients and healthy individuals. The mRNA and protein expression levels of IL-2 and IL-10 were also determined and compared between the GD patient and healthy control groups. In addition, the study aimed to determine whether the IL-2 and IL-10 expression levels may be used as biological markers of GD.

## Materials and methods

### Patients

A total of 256 individuals were enrolled into the study, including 118 patients with GD (female, 80; male, 38; mean age, 42.7±11.2 years) and 138 healthy individuals (female, 89; male, 49; mean age, 41.8±10.8 years). The GD patients had been newly diagnosed with GD and had not received any therapies prior to participation in this study. The healthy individuals did not possess antithyroid autoantibodies or a family history of autoimmune disorders and served as the control group. As demonstrated in Table I, no statistically significant differences were detected in the age and gender distribution between the GD and healthy groups (P>0.05). All the experiments were conducted according to the Ethical Guidelines of the Linyi People’s Hospital (Linyi, China) and written informed consent was obtained from all the participants. The present study was approved by the Ethics Committee of Linyi People’s Hospital (Linyi, China).

### Enzyme-linked immunosorbent assay (ELISA)

Blood samples (1 ml) were collected from all the patients and healthy individuals, and were centrifuged at 800 × g for 15 min for serum separation. The serum samples were analyzed for specific antibodies using an indirect ELISA method, as described in a previous study (18). GD patient serum samples were defined as the GD group, whereas serum samples of the healthy controls were defined as the healthy group. Goat anti-mouse horseradish peroxidase (HRP)-labeled immunoglobulin (Ig)G (1:2,000; Santa Cruz Biotechnology, Inc., Dallas TX, USA) was used as the secondary antibody. The absorbance at 450 nm was measured using a microplate reader (Multiskan MK-3; Thermo Fisher Scientific, Vantaa, Finland). The antibody titers were calculated as the highest dilution providing a positive reading. The cut-off value was set to double the mean absorbance detected in the serum samples of the negative healthy controls.

### Reverse transcription-quantitative polymerase chain reaction (RT-qPCR)

Total RNAs were harvested from the peripheral blood mononuclear cell samples using the RNeasy kit (Qiagen Inc., Valencia, CA, USA), according to the manufacturer’s instructions. The RT-qPCR experiments were repeated for a minimum of four times. Subsequently, the total RNA samples (1 μl) were reversely transcribed into cDNA using random primers in a Reverse Transcription II system (Promega Corporation, Madison, WI, USA), according to the manufacturer’s instructions. Next, the mRNA expression levels were determined by qPCR using an ABI Sequence Detection system (Applied Biosystems Life Technologies, Foster City, CA, USA). An assay reagent containing premixed primers and a VIC-labeled probe was used to determine the mRNA expression levels of endogenous GAPDH. The FAM fluorescent intensity was measured to detect the cDNA expression levels of IL-2 and IL-10, while the VIC fluorescent intensity was measured to determine the cDNA expression of endogenous GAPDH, using the ABI 7900HT Fast Real-Time PCR system (Applied Biosystems Life Technologies). The relative mRNA expression levels of IL-2 and IL-10 were normalized to the level of GAPDH mRNA of each individual. The primers used in RT-qPCR are listed in Table II.

### Western blot analysis

Peripheral blood mononuclear cells were collected for western blot analysis. Total proteins were isolated and separated using SDS-PAGE, followed by immunoblot analysis. The primary antibodies against IL-2, IL-10 and GAPDH were purchased from Santa Cruz Biotechnology Inc. The antibodies used in this study were as follows: rabbit polyclonal anti-IL-2 IgG antibody (cat no., sc-7896; 1:200); mouse monoclonal anti-IL-10 IgG2b antibody (cat no., sc-8438; 1:200); and goat polyclonal anti-GAPDH IgG antibody (cat no., sc-20357; 1:10,000). The secondary antibodies used in this study were the goat anti-mouse IgG-HRP (cat no., sc-2005; 1:10,000; Santa Cruz Biotechnology Inc.) and goat anti-rabbit IgG-HRP (cat no., sc-2004; 1:5,000; Santa Cruz Biotechnology Inc.) antibodies. The bound antibodies were detected using an enhanced chemiluminescence reagent (Pierce Biotechnology Inc., Rockford, IL, USA). The experiments were repeated for a minimum of three times. The obtained images were quantified using the Image Quant 7.0 software (GE Healthcare Life Sciences, Little Chalfont, UK).

### Restriction fragment length polymorphism (RFLP)

RT-qPCR was performed in a solution with total volume of 50 μl, which consisted of 200 ng of DNA templates isolated from the blood of the patients and healthy controls, 2 units Taq polymerase (Takara Bio Inc., Otsu, Japan) 200 μM deoxynucleoside triphosphate (Takara Bio Inc.), 1 pmol PCR primer, 1.5 mM MgCl_2_, 10 mM Tris-HCl (pH 8.3) and 50 mM KCl. In order to amplify the region of interest in the promoter of the IL-2 gene, the forward primer had a T base at the position −333, which was altered to C, creating a restriction site for the *Mae*I enzyme. The primers used are listed in Table II. RFLP analyses were conducted in a reaction volume of 20-μl using 1.5 units *Mae*I (New England Biolabs, Ltd., Hitchin, UK). The RFLP analysis of IL-10 was performed following a similar method, using the primers listed in Table II and the *Xag*I enzyme (New England Biolabs, Ltd.).

### Statistical analysis

The experimental data are expressed as the mean ± standard deviation. The SPSS statistical software (version 10.0: SPSS, Inc., Chicago, IL, USA) was used to perform independent sample t-tests, followed by one-way analysis of variance. In all the analyses, P<0.05 was considered to indicate a statistically significant difference. The correlation between the polymorphisms and risks of GD was indicated by the odds ratio (OR) and the 95% confidence interval (CI), which were calculated using a nonconditional logistic regression model, adjusting for ages, gender and other factors. The distribution of allele frequency was determined by Hardy-Weinberg equilibrium.

## Results

### Elevated serum levels of IL-2 and IL-10 in GD patients

**Figure 1:**
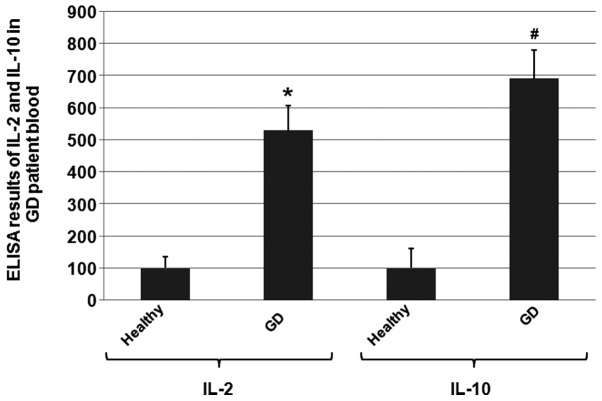
Expression levels of IL-2 and IL-10 in the blood of the GD patients and healthy controls, determined by ELISA. The data (mean ± standard deviation) were obtained from four independent experiments and calculated against the ratios of the reading obtained from the healthy control groups, which was set to 100. ^*^P<0.05, IL-2 vs. control; and ^#^P<0.05, IL-10 vs. control. IL, interleukin; GD, Graves’ disease.

To determine whether the serum levels of IL-2 and IL-10 were altered in the GD patients when compared with the healthy controls, blood (1 ml) was collected from all the patients and healthy individuals, followed by centrifugation for serum separation and determination of the serum levels using ELISA. As shown in Fig. 1, the ELISA results revealed a 5.2-fold and 7-fold increase in the IL-2 and IL-10 serum levels, respectively, when compared with the healthy controls. These results indicated that the IL-2 and IL-10 expression levels were altered in the GD patients.

### Upregulation of IL-2 and IL-10 mRNA expression levels in GD patients

To determine whether the increased IL-2 and IL-10 serum levels resulted from the upregulation of IL-2 and IL-10 mRNA expression levels, the mRNA transcript levels in all the patients and healthy controls were determined using RT-qPCR. The mean mRNA expression levels of IL-2 and IL-10 of the healthy individuals were assigned a value of 100.

**Figure 2:**
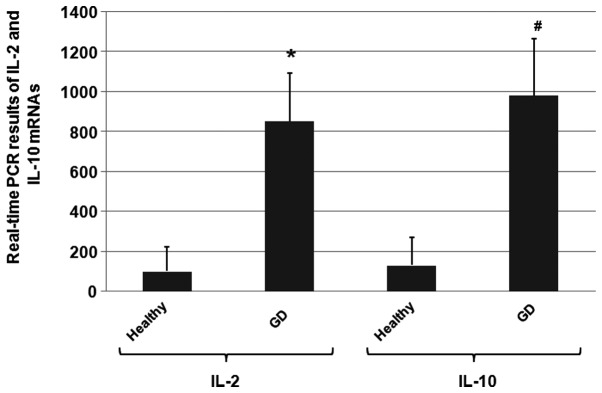
mRNA expression levels of IL-2 and IL-10 in GD patients and healthy controls, determined using reverse transcription-quantitative polymerase chain reaction. The relative amounts of IL-2 and IL-10 transcripts were normalized to the amount of GAPDH mRNA in the same individual. ^*^P<0.05, IL-2 vs. control; and ^#^P<0.05, IL-10 vs. control. IL, interleukin; GD, Graves’ disease.

As shown in Fig. 2, the mean mRNA expression levels of IL-2 and IL-10 in the blood samples of the GD patients were approximately 8-fold and 10-fold higher compared with the levels in the healthy control group (P<0.05). These results indicated that the IL-2 and IL-10 mRNA expression levels in GD patients were significantly increased when compared with the healthy individuals.

### Increased protein expression levels of IL-2 and IL-10 in the GD patients

**Figure 3:**
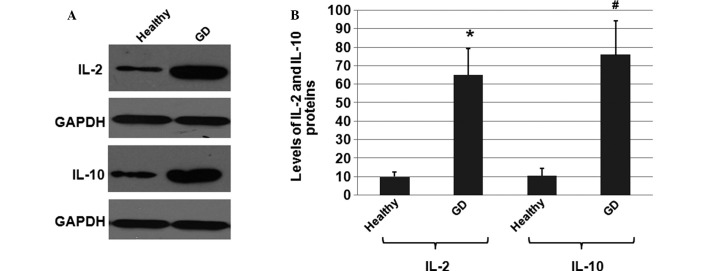
(A) Representative western blot, using GAPDH as the loading control, and (B) protein expression levels of IL-2 and IL-10 in the healthy control and GD patients. Total proteins were isolated and separated by SDS-PAGE and subjected to immunoblot analyses, using primary antibodies against IL-2, IL-10 and GAPDH. ^*^P<0.05, IL-2 vs. control; and ^#^P<0.05, IL-10 vs. control. IL, interleukin; GD, Graves’ disease.

To investigate whether the protein expression levels of IL-2 and IL-10 were altered in the GD patients when compared with the healthy individuals, total proteins were extracted from the healthy individual and GD patient samples. The IL-2 and IL-10 protein expression levels were determined using western blot analysis, with the cellular GAPDH protein used as the loading control. The mean normalized optical density (OD) values of the IL-2 and IL-10 protein bands relative to the OD values of the GAPDH bands from the same individual were calculated and subjected to statistical analysis (Fig. 3A).

Representative western blots from healthy individuals and GD patients are shown in Fig. 3A. As shown in Fig. 3B, the mean protein expression levels of IL-2 and IL-10 in GD patients were significantly higher compared with the levels in the healthy controls. A 6.4-fold increase was detected in IL-2 expression, while a 7.6-fold increase was detected in IL-10 expression, compared with the control group expression levels. These results indicated that the expression levels of the IL-2 and IL-10 proteins were significantly increased in the GD patients when compared with the healthy individuals.

### IL-2 -330T/G and IL-10 -1082A/G polymorphisms may be risk factors for GD in the Asian population

Polymorphisms at the −330 locus of the IL-2 promoter and the −1082 locus of the IL-10 promoter were analyzed in the GD patients and healthy controls. The allele frequency distribution of IL-2 at the −330 locus, and of IL-10 at the −1082 locus was in Hardy-Weinberg equilibrium (P>0.05; data not shown). Subsequently, putative correlations of the genotype distribution of the −330 IL-2 and −1082 IL-10 promoter loci with the occurrence rates of GD were investigated. As demonstrated in Table III, the increased number of GG single nucleotide polymorphisms (SNPs) in the GD group was detected in the two loci. These results indicated that the relatively high rates of homozygous GG SNPs on the alleles may be associated with the incidence of GD.

## Discussion

GD, an autoimmune disorder, is the cause of the majority of hyperthyroidism cases. Previous studies have demonstrated that GD is a genetically complex disease (19,20). Genetic predisposition to GD is based on multiple genes with limited individual effects. In the present study, variations in the expression levels of IL-2 and IL-10 between the healthy control and GD patient groups were investigated.

The ELISA results of the current study indicated that the IL-2 and IL-10 serum levels of the GD patients were increased by ~5.2-fold and ~7-fold, respectively, compared with the levels in the healthy controls. These observations were supported by the RT-qPCR and western blot results. The RT-qPCR results indicated that the IL-2 and IL-10 mRNA expression levels were increased in GD patients when compared to the healthy controls. In addition, the western blot analysis results demonstrated that the protein expression levels of IL-2 and IL-10 were significantly increased in the GD patients. These results indicated that the development of GD is associated with the alterations in the IL-2 and IL-10 gene expression levels.

Alterations in gene expression levels frequently result from alterations in gene promoter activities (21). The RFLP results of the present study indicated that an increased number of GG SNPs in the GD group was identified in the −330 locus of the IL-2 promoter and the −1082 locus of the IL-10 promoter. These results indicated that the relatively high rates of homozygous GG SNPs on the alleles may be associated with the development of GD. Therefore, SNPs may serve as biomarkers in GD patients, at least in the Asian population.

Recently, IL-16 polymorphism has been demonstrated to be associated with GD in the Taiwanese population (22), using sliding-window haplotype analysis. The authors demonstrated that the most significant haplotype was provided by the 6-SNP haplotype window, including rs7182786, rs8028364, rs12907134, rs4128767, rs4072111 and rs8031107, indicating that IL-16 may be a genetic marker for the diagnosis and prognosis of GD in the Taiwanese population (22). In addition, an association between IL-10 polymorphism and Graves’ disease has been reported in the Turkish population (23). A recent meta-analysis of 11 case control studies indicated that polymorphisms at the −511 locus of IL-1B are associated with the risk of GD development in Caucasian and Asian individuals in a homozygotic model (24). SNPs of the IL-21 gene have been found to be associated with development of GD (25), while individuals with SNPs at the common IL-21 and IL-21R genes may present a higher risk of developing Hashimoto’s thyroiditis (25). However, interleukin gene polymorphisms are not only associated with GD. The association between interleukin gene polymorphisms and other diseases have also been investigated. For instance, a previous study demonstrated that the −590 C/T polymorphism of IL-4 promoter is associated with genetic susceptibility to rheumatoid arthritis (26). The results of the present study facilitate the understanding of the association between interleukin gene polymorphisms and the diagnosis and prognosis of GD.

In conclusion, the results of the present study demonstrated that patients with GD have significantly higher levels of IL-2 and IL-10, and significantly higher rates of homozygous GG SNPs (IL-2 -330T/G and IL-10 −1082A/G polymorphisms). These findings suggest that IL-2 and IL-10 may be potential biomarkers for the incidence of GD.

## Figures and Tables

**Table I:** Information of GD patients and healthy individuals.

Parameter	Healthy individuals (n=138)	GD patients (n=118)
Age (years)	36–52	35–54
Mean ages (years ± SD)	41.8±10.8	42.7±11.2
Gender, female/male (n)	89/49	80/38
Severe	-	81
Highly severe	-	37

GD, Graves’ disease.

**Table II:** Primers used in this study.

Primers	Primer sequences	Targets
IL-2_F	5′-ATGGTAACTACCGCGTATTGCA-3′	IL-2
IL-2_R	5′-GTACCTAGGCTTTATCGATTCG-3′	
IL-10_F	5′-CTTCGAGATCTCCGAGATGCCTTC-3′	IL-10
IL-10_R	5′-ATTCTTCACCTGCTCCACGGCCTT-3′	
GAPDH_F	5′-TGGCTATTGCATGTTCAACCA-3′	GAPDH
GAPDH_R	5′-GTCGAAGGTGAACTGTGTTCCT-3′	
RFLP_IL-2_F	5′-TATTCACATGTTCAGTGTAGTTCT-3′	IL-2
RFLP_IL-2_R	5′-CATTGTGGCAGGAGTTGAGGT-3′	
RFLP_IL-10_F	5′-CCAAGACAACACTACTAAGGCTCCTTT-3′	IL-10
RFLP_IL-10_R	5′-GCTTCTTATATGCTAGTCAGGT-3′	

IL, interleukin; RFLP, restriction fragment length polymorphism F, forward; R, reverse.

**Table III:** Association of SNPs at the -330 locus of IL-2 promoter and the -1082 locus of IL-10 promoter with the risk of GD development.

Sites	SNPs	Healthy individuals, (n=138), n (%)	GD patients, (n=118), n (%)	P-value^a^	OR (95% CI)
IL-2	TT	114 (82.6)	92 (77.9)		
-330	TG	24 (17.4)	18 (15.4)	0.059	1.603 (0.242–0.671)
	GG	0 (0)	8 (6.7)	0.043	1.104 (1.821–2.882)
IL-10	AA	87 (63.0)	74 (62.7)		
-1082	AG	49 (35.5)	37 (31.3)	0.051	1.271 (0.562–3.269)
	GG	2 (1.5)	7 (5.9)	0.028	1.214 (1.321–3.645)

a Non-conditional logistic regression analysis was adjusted by using multiple factors including gender and ages.

SNP, single nucleotide polymorphism; GD, Graves’ disease; IL, interleukin; OR, odds ratio; CI, confidence interval.
